# The predictive effects of AI anxiety on 21st-century skills and lifelong learning tendencies: a study of pre-service teachers in Northern Cyprus

**DOI:** 10.3389/fpsyg.2026.1810689

**Published:** 2026-05-13

**Authors:** Çiğdem Cantaş, Cevat Celep, Özgür Batur

**Affiliations:** 1 Department of Educational Administration, Supervision and Planning, Faculty of Education, Girne American University, Kyrenia, Cyprus; 2 School of Foreign Languages, Final International University, Kyrenia, Cyprus; 3 Department of Primary School Teaching, Faculty of Education, Girne American University, Kyrenia, Cyprus; 4 Department of Educational Sciences, Final International University, Kyrenia, Cyprus

**Keywords:** 21st-century skills, AI anxiety, educational psychology, higher education, lifelong learning, pre-service teachers

## Abstract

**Introduction:**

The introduction of artificial intelligence (AI) into education presents a significant psychological challenge for students, potentially eliciting specific anxieties that may influence the development of 21st-century competencies and lifelong learning tendencies. This study examines the predictive effects of AI anxiety dimensions (Learning AI, Job Replacement, Sociotechnical Blindness, and AI Configuration) on 21st-century skills and lifelong learning tendencies among pre-service teachers in Northern Cyprus.

**Methods:**

Using a quantitative design, data were collected from 396 pre-service teachers enrolled in education faculties. Validated scales for AI Anxiety, Multidimensional 21st-century Skills, and Lifelong Learning were administered. Data were analyzed using multiple regression analyses to determine the predictive power of specific AI anxiety dimensions on distinct skill domains.

**Results:**

While overall AI anxiety did not predict 21st-century skills, specific dimensions showed selective predictive power. Learning AI anxiety was a significant negative predictor of Critical Thinking, Problem-Solving, and Career Awareness. Job Replacement anxiety was a significant negative predictor of Social Responsibility and Leadership. Conversely, Sociotechnical Blindness emerged as a significant positive predictor of Social Responsibility and Leadership. The AI Configuration dimension and lifelong learning tendencies were not significantly predicted by these anxieties.

**Discussion:**

The findings indicate that AI anxiety is multidimensional and affects specific 21st-century skill domains selectively. Because lifelong learning orientations remained stable, educational interventions should move beyond broad AI literacy and instead target specific psychological concerns, such as learning-related anxiety and fears regarding job replacement, to better support future educators.

## 1 Introduction

The emergence of Artificial Intelligence (AI) within educational ecosystems has created a radical paradigm shift, restructuring learning environments and skill acquisition while simultaneously redefining broader societal frameworks of the digital era (Gao et al., 2024). The integration of AI, including generative platforms such as ChatGPT (Generative Pre-trained Transformer), adaptive learning systems, automated tutoring, personalized learning, and remote assessment, has fundamentally changed how students develop core competencies (Asy'ari and Sharov, 2024; Bhatia et al., 2024). In this regard, students are expected to acquire higher-order 21st-century skills that include critical thinking, creativity, collaboration, and flexibility, along with a strong commitment to participate in lifelong learning (Benvenuti et al., 2023; Polat, 2025; Wang and Li, 2024).

While the integration of AI technology revolutionizes the educational process and provides academic advantages, several pedagogical and psychological challenges have emerged. A significant challenge associated with the implementation of AI in the education sector is the emergence of AI anxiety, defined as fear and anxiety concerning the usage, implication, and potential outcomes of AI technologies (Lin and Chen, 2024; Zhan et al., 2024). This anxiety has been identified as an influential yet under-explored factor shaping students' academic emotions, motivational attitudes, and their sustained readiness to engage in lifelong learning within the rapidly changing digital world (Almaiah et al., 2022; Çelik et al., 2024; Hwang and Wu, 2025).

This study investigates the impact of pre-service teachers' AI anxiety on the development of 21st-century skills and lifelong learning tendencies by grounding its analysis in (Wang and Wang 2019) framework, Sweller's (1988) Cognitive Load Theory (CLT), (Deci and Ryan 1985) Self-Determination Theory (SDT), and Bandura's (1977) Social Learning Theory (SLT). (Wang and Wang 2019) identified four core dimensions of AI anxiety—Learning AI, Job Replacement, Sociotechnical Blindness, and AI Configuration—providing a framework to examine the impact of AI anxiety on students' 21st-century competencies and lifelong learning behavior. According to CLT, learning is most effective when the mental burden on working memory is optimized, providing a critical construct for understanding how the technical complexity of AI configuration can create an extraneous cognitive load that diverts resources from higher-order skill acquisition (Baxter et al., 2025; Kalyuga and Sweller, 2004; Ward et al., 2020). SDT posits that anxiety impacts the motivation to learn by negatively affecting students' perceptions of their capacity to fulfill their basic psychological needs of autonomy and competence (Deci and Ryan, 2000; Wang et al., 2025). Specifically, SDT postulates that students who perceive AI as a threat to job security (job replacement) develop an existential threat (Li et al., 2025), which undermines their motivation to partake in learning activities that involve self-directed behavior, such as entrepreneurial activities and leadership development (Meletiadou et al., 2025). SLT assumes that students can develop 21st-century skills and attitudes toward lifelong learning by observing and imitating AI behavior; therefore, AI anxiety and its dimensions may act as affective inhibitors that can hinder the cognitive processes of attention and self-efficacy needed to model successfully (Bandura and Walters, 1963). However, while these inhibitors may disrupt context-specific skill acquisition, it is hypothesized that more stable, self-determined orientations such as lifelong learning may remain unaffected due to their intrinsic nature. These theories offer an integrated conceptual understanding that helps clarify how motivational processes influence the development of cognitive abilities and adaptive skills in the context of pre-service teachers' AI anxiety. (Wang Y. M. et al. 2024) point out that these aspects of AI anxiety are interlinked and need stronger empirical research. For instance, in a study of secondary school students studying English as a foreign language, social influence, and facilitating conditions were identified as positive predictors of intentions to learn with AI, while AI anxiety was a significant negative predictor of behavioral intention (Wen et al., 2024). These results suggest that anxiety's role in shaping adoption intentions is not limited to direct effects; instead, it has multifaceted mental processes that influence how students perceive the utility and accessibility of AI technologies.

The significance of this topic stems from the dual impact of AI on student development. The use of AI has already demonstrated the capacity to stimulate creativity, strengthen student engagement, and provide personalized feedback, which are integral to the development of 21st-century competencies and positive academic affect (Çelik et al., 2024; Gao et al., 2024; Lin and Chen, 2024). Furthermore, empirical studies demonstrate that applications based on AI, such as ChatGPT, have been found to reduce certain negative affective states, including test anxiety, and offer support on academic wellbeing (Gao et al., 2024).

Research has also shown that AI anxiety indicators differ considerably among various demographic groups. University students who have been exposed to AI-mediated language-learning systems have reported anxiety, particularly regarding their writing abilities. As research notes, increased reliance on AI does not necessarily decrease anxiety levels; in fact, it can even increase these levels as students start to rely more on the technology (Kaya and Çelebi, 2025; Yu, 2024). This aligns with the Yerkes-Dodson Law whereby low anxiety levels enhance students' overall performance (Batool et al., 2025; Eysenck et al., 2007). However, repetitive and depersonalized AI interactions, ongoing algorithmic assessment, and technical aggravations can inhibit creativity, lead to emotional disconnection, and increase performance anxiety. These challenges are further augmented by the threat of overreliance on AI, which may undermine independent thinking, self-efficacy, and challenge the ability of students to independently resolve problems (Rodríguez Bermeo et al., 2025; Zhan et al., 2024).

Recent empirical studies have begun to show the subtle impact of AI on students' academic emotions, motivations, and skill progress. The literature shows a pattern where digital efficacy, confidence in using AI, and the positive emotional experience of students are the main predictors of a successful acquisition of skills and a desire for lifelong learning (Ahn, 2024; Çelik et al., 2024; Wang and Li, 2024). Based on this, (Cengiz and Peker 2025) showed that AI anxiety is not an independent barrier but a part of a sequential mediating relationship in which literacy and internal attitudes determine the level of acceptance or fear of generative AI. However, the impact of AI on higher-order cognitive abilities, such as creativity and critical thinking, remains unclear, with some studies reporting that AI has a minimal or even negative impact on autonomous cognition and problem solving (Jia and Tu, 2024; Lin and Chen, 2024; Rodríguez Bermeo et al., 2025; Zhang et al., 2025).

Although there is increasing research, the theoretical and empirical gaps between selective effects of AI anxiety dimensions on particular skill domains remain understudied. AI anxiety represents a complex psychological phenomenon and can be described as a combination of anxiety-provoking, uncomfortable, and stressful reactions that occur as a result of encountering AI technology (Çoban et al., 2025; Ilhan, 2025; Wang and Wang, 2019). A common perspective found in the literature has been developed from earlier computer anxiety studies and classical cognitive performance theories. This perspective defines an overarching model based on the idea that general levels of anxiety negatively affect students' academic performance and skill development in a way that is generally applicable across all areas. The basis for this belief can be seen in many of the early works of cognitive psychology, including the Processing Efficiency Theory (Eysenck and Calvo, 1992), which states that worry, the cognitive element of anxiety, uses up available working memory space, thereby reducing processing efficiency for all cognitive tasks with a certain level of demand (Sarason, 1988). Similar to these cognitive works, other early studies of computer anxiety have shown a direct inverse relationship between generalized anxiety, generalized learning avoidance, and overall poor skills development (Harrington et al., 1990; Torkzadeh and Angulo, 1992). However, there is growing evidence refuting this assumption, suggesting that anxiety has differential effects across various skill types (Chen et al., 2024; Daker et al., 2023; Hasan et al., 2025; Liu et al., 2024; Pacheco et al., 2025). Moreover, the instructional climate also conditions these outcomes; as (Hazzan-Bishara et al. 2025) argue, educational support and information credibility may help strengthen self-efficacy among teachers, thus establishing a classroom setting that will help either alleviate or enhance student anxiety about AI.

In addition, this study fills a critical gap in the literature by explicitly differentiating between Lifelong Learning Tendency (LLT) and general skills of the 21st-century. In the context of the SDT and LLT is conceptualized as a stable need-satisfaction-based intrinsic motivational tendency (Åström et al., 2025; Shevchenko et al., 2025). This inherent basis indicates that LLT is motivationally more resilient to the extrinsic threats of AI anxiety than 21st-century skills are, which are contextually susceptible, a pattern consistent with theoretical research on how internal motivation affects foreign language anxiety (Nyuhuan, 2024). Parallel to this, the findings of test and foreign language anxiety research support the domain-specific nature of the effects of apprehension (Hasan et al., 2025; Li, 2022). Although previous research has found a complex association between AI anxiety and the acquisition of modern skills, reporting that anxiety can enhance effort expectancy (Lemay et al., 2020; Wang et al., 2019) and, at the same time, interfere with technological acceptance and pedagogical results (Schiavo et al., 2024; Susanti, 2025), the literature does not provide a systematic study of these heterogeneous effects. By integrating CLT and SDT, this study offers a theoretically grounded explanation of how anxiety influences student development in AI-augmented learning settings.

## 2 Theoretical framework

### 2.1 AI anxiety

Artificial intelligence anxiety is a multi-faceted psychological condition, which involves fear, nervousness, and stressful reactions that prevent individuals from engaging with AI technologies, their implications, and perceived outcomes (Uçar et al., 2024). AI anxiety extends beyond mere technology discomfort to existential questions of human identity, economic stability, and social change (Cengiz, 2025). Understanding AI anxiety as multidimensional rather than a single unified fear is essential to capturing its varied influence on student development. The present study is based on the four-component framework developed by (Wang and Wang 2019), and is supported by recent empirical studies.

Learning AI refers to concerns related to the knowledge and skills required to effectively use AI systems (Sha et al., 2024). The dimension captures the unease that individuals feel when they become acquainted with AI methods, applications, and theoretical principles. This anxiety is generally non-linear, whereby the initial exposure is often shaped by misconceptions; subsequent engagement can transform this anxiety into more complex concerns regarding the ethical and societal consequences of the technology. Research has shown that even though students might experience a moderate level of general anxiety, their specific concerns regarding the learning process are a major predictor of their overall AI self-efficacy (Asio and Suero, 2024). Learning AI anxiety in the educational setting manifests as the unwillingness to use new tools, which directly affects the reproduction stage of observational learning, as anxiety suppresses both the physical readiness and cognitive capacity needed to reproduce a modeled skill (Bandura, 1986).

Job Replacement anxiety describes concerns about the replacement of human labor by AI systems (Uçar et al., 2024). This dimension consistently ranks among the highest reported scores across various populations, particularly among pre-service and in-service teachers (Yang, 2024). Although Job Replacement anxiety is primarily regarded as a negative stressor, it can also act as a potent extrinsic motivator. For many, the fear of being replaced by automation can create a compelling incentive to improve job performance and acquire AI-related competencies to maintain their career prospects.

Sociotechnical Blindness refers to fear stemming from the perception that AI operates beyond human control or understanding (Johnson and Verdicchio, 2017). (Ugur and Dursun 2025) state that it involves concerns regarding understanding broader social, ethical, and systemic implications. This phenomenon causes agitation, as individuals cannot predict or influence the outcomes of the systems they use. Sociotechnical Blindness is a threat to autonomy and relatedness, as it leaves humans feeling marginalized by computer autonomy (Croitoru et al., 2025). According to recent empirical research related to individuals' limited awareness of how AI systems are socially embedded, sociotechnical blindness emerged as a structurally influential element of AI anxiety (Qiu et al., 2025; Ugur and Dursun, 2025; Zhang et al., 2025).

AI Configuration focuses on anxiety linked to algorithmic bias, technical setup, maintenance and integration of AI systems (Çoban et al., 2025). Research into the ethical and pedagogical risks related to AI in education suggests that concerns such as ethical use, accountability, and integration barriers are prevalent among educators and learners, directly influencing technology acceptance and contributing to AI anxiety (Cengiz and Peker, 2025; Filiz et al., 2025; Klimova and Pikhart, 2025), potentially diminishing academic achievement (Batool et al., 2025), and collaborative learning (Fan et al., 2025). Studies based on the CLT (Sweller, 2011) have demonstrated that task-irrelevant complexity, such as ineffective interface design or distracting features, augments extraneous cognitive load, thereby distracting working memory from the main learning activity toward the management of the technology itself (Skulmowski and Xu, 2022). Additionally, empirical studies show that anxiety is also linked to increased extraneous cognitive load (Fredericks et al., 2021), suggesting that anxiety caused by technological setup can also deplete the cognitive resources required for deep learning.

### 2.2 Conceptualization of 21st-century skills

Twenty-first-century skills have been conceptualized using a variety of international institutional and scholarly frameworks, which, although they may vary in terms of terminology and focus, exhibit significant conceptual overlap. Influential frameworks such as the Partnership 21st-century Skills, the OECD and the Assessment and Teaching of 21st-century Skills initiative continue to emphasize the combination of cognitive, technological, social, and self-regulatory skills that are essential for effective engagement in the modern academic, professional, and civic environment (Ananiadou and Claro, 2009; Binkley et al., 2012; National Research Council, 2011; OECD, 2023). These frameworks collectively emphasize critical thinking, digital literacy, teamwork, adaptability, ethical responsibility, and lifelong learning as essential capacities for navigating rapid technological changes. Based on this convergent view, the current study adopts a focused operationalization of 21st-century skills that is conceptually aligned with AI-mediated learning contexts and empirically adequate to investigate the role of AI anxiety.

Competencies vital for academic achievement and labor readiness in fast-changing global environments, known as 21st-century skills, are grouped into five dimensions (Larson and Miller, 2011; Çelik et al., 2024; Yuanti et al., 2025). The first, Information and Technology Literacy Skills (ITLS), refers to the ability to effectively locate, evaluate, utilize, and communicate information through digital means. This dimension extends beyond basic computer operation to include critical evaluation of information sources and comprehension of AI systems' capabilities and limitations (Salhab and Aboushi, 2025). Critical Thinking and Problem-Solving Skills (CTPS) are cognitive abilities that allow individuals to examine and assess information and find a rationale for solving non-routine problems, which is especially essential in exploring AI-generated content. Entrepreneurship and Innovation Skills (EIS) refer to the capacity to identify opportunities, devise new solutions, take rational risks, and apply a creative approach not only in the realm of traditional business but in all fields of work (Yuanti et al., 2025). Social Responsibility and Leadership Skills (SRLS) refer to skills that allow individuals to collaborate toward a common purpose and remain ethically committed to the greater societal wellbeing, particularly in relation to the ethical application and systemic influence of AI (Ram, 2025). Career Awareness (CA) includes cognitive competencies that allow individuals to comprehend various career choices, evaluate their strengths, and create strategic career plans, which are crucial for a labor market characterized by technological disruption and job displacement issues (Yaşar and Karagucuk, 2025).

### 2.3 Implications for lifelong learning tendency

In today's technological environment, LLT has become increasingly critical to career adaptability and resilience (Karatas et al., 2021). LLT is defined as students' willingness to engage autonomously in learning (Ryan and Deci, 2024). SDT provides a direct theoretical mechanism for understanding the link between AI anxiety and long-term learning: students' motivation to pursue LLT is dependent on their fulfillment of the psychological needs for competence and autonomy. Job Replacement anxiety, in particular, threatens competence by suggesting that current skills are obsolete and jeopardize autonomy by implying that career paths are externally controlled by technology (Wang K. et al., 2024). Consequently, the extent to which LLT is predicted may depend on the degree to which AI anxiety impairs core motivational needs. Recent research on adult and non-traditional learners suggests that anxiety arising from rapid AI-driven technological change and misinformation can discourage re-engagement with education, thereby posing an additional contextual barrier to sustained lifelong learning (Barrett and Miller, 2025). However, the SDT framework also suggests that LLT, being intrinsically motivated, may be structurally resilient to external, fear-based technological threats, differentiating its predictive pathway from situation-specific skill acquisition (Chen et al., 2024). Students with higher self-efficacy may transform the negative affective state of anxiety into a challenge, thereby acquiring new skills and maintaining their propensity for long-term learning (Varol, 2025).

### 2.4 Cognitive load theory

CLT states that working memory has a finite capacity and that learning is most effective when the mental burden on working memory is optimized (Sweller et al., 2011; Zheng, 2017). Cognitive load refers to the mental effort required to process information and perform learning activities within the constraints of working memory (Sweller, 2018). As these cognitive resources are limited, effective instructional design is essential to prevent unnecessary mental burden. In AI-integrated learning environments, anxiety related to understanding, configuring, or interacting with AI systems may introduce additional cognitive load, thus distracting cognitive resources necessary to engage in deep processing and schema construction. This distraction prevents the development of more advanced skills, such as critical thinking, information literacy, and problem solving, especially when advanced cognitive reactions are most needed (Lemay et al., 2021). In this context, CLT provides a useful framework for investigating how AI-related anxiety influences students' mental effort and performance outcomes. However, more refined models consider the fact that the association between anxiety and skill development exhibits non-linear characteristics. Aligning with the Yerkes-Dodson Law, this study considers that although high levels of anxiety are inhibitory, moderate levels may stimulate motivation and engagement, provided they are accompanied by a sufficient level of self-efficacy and social support (Chen et al., 2024). This non-linear relationship suggests an inverted-U shaped function, whereby some level of concern stimulates engagement, but high levels of anxiety inhibit the activity.

### 2.5 Social learning theory

SLT developed by Albert Bandura, explains how individuals learn behaviors, attitudes, and skills by observing and imitating others. SLT suggests that the process of learning new competences is a cognitive process that includes attention, retention, reproduction, and motivation (Bandura, 1986), which provides a critical lens to observe how AI anxiety affects the development of skills and dispositions associated with lifelong learning. For effective observational learning, students need to first focus their attention on important aspects of the modeled behavior, such as an instructor or a peer using generative AI to solve complicated problems. However, high AI anxiety levels, especially within the “Learning” and the “Configuration” dimensions, may be cognitive distractors which can interfere with the attention stage and cause mental attention to shift away from the skill being modeled to the apparent complexity or threat of the tool.

Moreover, SLT also points out that learning and performance are distinctive; individuals may be knowledgeable but not necessarily perform a successful reproduction. Anxiety can inhibit performance despite cognitive understanding, often through reduced self-confidence or affective interference that constrains action. Students who have high AI anxiety may underestimate their expressive and creative abilities relative to external assessments, and thus constrain their performance of observed 21st-century skills (Ouyang, 2025). On the other hand, a supportive social environment offers vicarious reinforcement, where others who have ethically and successfully implemented AI in their workflows can motivate the observer to follow suit. In the digital age, modeling is no longer confined to the human models but rather extends to the symbolic models, including AI-based content and virtual assistants, which represent the tools of algorithmic socialization, that students have incorporated to internalize the dispositions of the 21st-century. Lastly, the principle of reciprocal determinism holds that the personal factors of the student (AI anxiety levels), the AI-enriched environment, and the actual behaviors are constantly influencing each other, that is, positive social interactions may buffer negative affective states and create the drive to engage in lifelong learning (Usher and Pajares, 2008).

### 2.6 Self-determination theory

SDT offers a motivational explanation, according to which the sense of intrinsic drive and psychological wellbeing depends on the accomplishment of the three fundamental psychological needs—autonomy, competence, and relatedness (Ryan and Deci, 2000). Job Replacement Anxiety directly infringes on the need to feel competent and autonomous (Huang and Zhao, 2025) because the threat of becoming obsolete in terms of skills cultivates a sense of personal incompetence or loss of control (Wang Y. M. et al., 2024). As a result, lack of intrinsic motivation decreases the inclination of individuals to participate in proactive self-determined actions including creative problem-solving and entrepreneurial activities. Furthermore, Sociotechnical Blindness anxiety may stem from confusion about autonomy and inaccurate perceptions of technological development, causing users to feel like passive observers rather than active agents in the decision-making process (Johnson and Verdicchio, 2017).

### 2.7 Social cognitive theory

**Figure 1:**
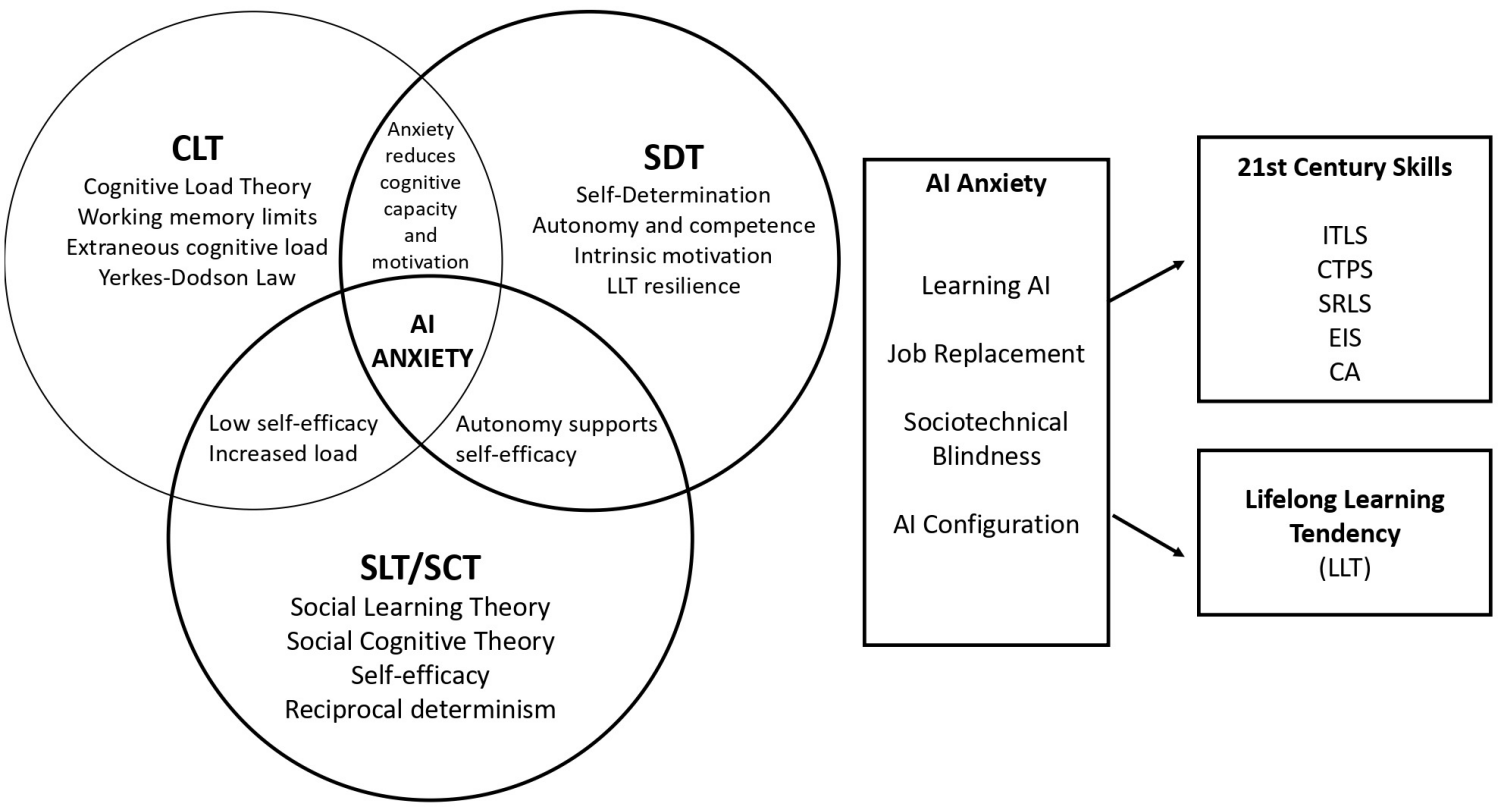
Conceptual framework of AI anxiety and learner development. This figure presents the theoretical overlap between Cognitive Load Theory (CLT), Self-Determination Theory (SDT), and Social Learning/Cognitive Theory (SLT/SCT). It maps the multidimensional nature of AI anxiety and its interaction with 21st-century skills and lifelong learning tendencies (LLT), providing the theoretical basis for the study's predictive regression model.

Social Cognitive Theory (SCT) is essential for contextualizing the relationship between anxiety and skill acquisition, as it identifies the critical role of self-efficacy—defined as the belief in one's ability to successfully perform behaviors (Bandura, 1997). In the context of AI-mediated environments, AI self-efficacy has a central role in shaping how students deal with AI anxiety. According to (Lazarus and Folkman 1984), having strong self-efficacy beliefs encourages individuals to perceive AI-related challenges as manageable and controllable and, thus consider the anxiety-provoking circumstances as challenges rather than threats. Persistence, experimentation, and proactive engagement with tasks are all promoted by this appraisal process and thus transform anxiety into a motivational source rather than behavioral avoidance. In contrast, students with low self-efficacy tend to view AI related demands as threatening, thereby increasing anxiety levels. Anxiety directly decreases the level of technology self-efficacy, leading to predictable behavioral avoidance of AI-related activities (Compeau and Higgins, 1995). The principle of reciprocal determinism by Bandura explains how the interaction between personal (e.g., AI anxiety and self-efficacy), environmental (e.g., AI system complexity and feedback), and behavioral (e.g., skill practice or disengagement) factors mutually influence each other over time (Bandura, 2001). These processes can lead to establishing a long-term learning path through either skill development or losing interest in AI-assisted learning due to initial differences in anxiety and self-efficacy. Collectively, these theories form a model which highlights the challenges of AI anxiety and its impact on developing skills and lifelong learning tendencies. The conceptual framework of the overlap between CLT, SDT, and SCT and their predictive relationship with the variables of the study is shown in Figure 1.

### 2.8 Synthesis and research gap

Reviewed literature suggests that AI anxiety is a complex phenomenon with psychological and behavioral effects (Wang and Wang, 2019). Although 21st-century skills and lifelong learning are well-studied fields, a critical and timely gap exists. Research on how distinct dimensions of AI anxiety predict different categories of 21st-century skills and lifelong learning tendencies remains limited. Current studies have tended to study AI-related attitudes or anxiety in aggregate, rather than analyzing how distinct dimensions of AI anxiety differentially predict particular 21st-century skills and lifelong learning tendencies.

## 3 The present study

Addressing this gap, the present study examines the extent to which specific dimensions of AI anxiety predict pre-service teachers' 21st-century skills and lifelong learning tendencies. By exploring these interconnections through the lenses of CLT, SDT, SLT, and SCT, the study seeks to provide empirical evidence on the different psychological barriers that influence the development of core competencies. Based on the theoretical framework presented, the study formulates the following hypotheses:

H1: AI anxiety significantly and negatively impacts students' 21st-century skills.

H2: AI anxiety significantly impacts students' lifelong learning tendencies.

H3: The dimensions of AI anxiety differentially impact specific 21st-century skill domains.

Empirical testing of these hypotheses aims to understand the mechanisms that affect students' responses to AI and the influence this has on skill acquisition and learning tendencies, ultimately informing the development of interventions to improve students' adaptive abilities within AI-augmented educational settings.

## 4 Methodology

### 4.1 Research design

The quantitative design in the study was cross-sectional, as it aimed at examining the predictive relationships between the sub-dimensions that constitute AI anxiety, 21st-century skills, and lifelong learning tendencies among pre-service teachers. Linear and multiple regression analyses were used to assess the predictability of independent variables on the dependent variables, which are appropriate techniques for evaluating the contribution of individual predictors to outcome measures (Creswell and Guetterman, 2018). To determine the generalized effect of AI anxiety on broad student outcomes, H1 and H2 used aggregate scores of AI anxiety, whereas the multidimensional nature of the construct was later tested in H3 to determine domain-specific differences, as postulated by the (Wang and Wang 2019) framework.

### 4.2 Study population and participants

The study group consisted of 1,827 undergraduate students who pursued their studies in four universities with education faculties in the Kyrenia region of the Turkish Republic of Northern Cyprus (TRNC). While all four institutions recruit international students, the undergraduate education departments from which the sample was drawn predominantly comprise local Turkish Cypriot and Turkish students, with a small number of international students concentrated in the English Language Teaching Program. This sample composition therefore reflects the disciplinary profile of undergraduate education departments in the region rather than the broader international intake of the institutions. A total of 396 participants voluntarily took part in the study in the 2023–2024 spring semester. The convenience sampling method, a non-probability approach, was adopted due to its practicability and frequent use in educational research (Cohen et al., 2000). To verify the adequacy of the sample size (*N* = 396), a priori power analysis was conducted using G^*^Power 3.1.9.7 (Faul et al., 2009) for a multiple regression with four predictors. With α = 0.05 and power (1–β) = 0.80, the sample was sufficient to detect a small-to-medium size effect size (Cohen, 1988), confirming the sample was adequately powered to detect the predictive relationships reported.

The sample was diverse in terms of gender, age, nationality, institution, academic program, and year of study. Most respondents were women (61.1%) and between the ages 21 and 23 (42.4%). In terms of nationality, 76.8% were from Turkey, 15.9% were from the TRNC, and 7.3% from other countries. The most common programs included English Language Teaching (45.7%), Preschool Education (21.7%), and Special Education Teaching (14.6%), and students were spread between the first to fourth years.

### 4.3 Data collection tools

To operationalize the study variables, three self-report scales were employed which had been previously tested and validated using established Turkish adaptations, ensuring cultural and linguistic relevance. These instruments were selected for their strong psychometric foundations and their direct relevance to the study constructs. The survey was conducted in both Turkish and English to accommodate international and domestic students.

#### 4.3.1 AI Anxiety Scale

The AI Anxiety Scale was originally developed by (Wang and Wang 2019) and adapted into Turkish by (Akkaya et al. 2021). The scale consists of 16 items distributed across four dimensions: Learning AI (e.g., “Learning to use AI techniques/products makes me anxious”), Job Replacement (e.g., “I am afraid that AI techniques/products will replace someone's job”), Sociotechnical Blindness (e.g., “I am afraid that an AI technique/product may be misused”), and AI Configuration (e.g., “I find humanoid AI techniques/products intimidating”). The confirmatory factor analysis (CFA) from the adaptation study validated the four-factor structure with good fit indices (χ^2^/df = 2.627, CFI= 0.950, and RMSEA = 0.078). Responses were collected on a five-point Likert scale. The scale has been widely used with consistent reliability (Hopcan et al., 2024; Wang Y. M. et al., 2024). In the present study, the scale showed a high level of internal consistency (Cronbach's α = 0.95).

#### 4.3.2 Multidimensional 21st-Century Skills Scale

Students' 21st-century skills were assessed with The Multidimensional 21st-Century Skills Scale developed by (Çevik and Sentürk 2019). This 41-item instrument is measured on a five-point Likert scale (1 = Strongly Disagree to 5 = Strongly Agree) and is organized into five dimensions: Information and Technological Literacy Skills (ITLS; e.g., “I access the needed information from reliable sources”), CTPS (e.g., a reverse-coded item: “I believe that every piece of information I am told is correct”), EIS (e.g., “I produce and apply new and useful ideas that go beyond the norm”), SRLS (e.g., “I often take on the role of a leader in group activities”), and Career Awareness Skills (CAS; e.g., “I strive to successfully complete the task given to me”). The original scale's development study confirmed the five-factor structure through CFA, reporting good fit indices (χ^2^/df = 2.73, CFI = 0.90, GFI = 0.91, and RMSEA = 0.06) and good reliability (Cronbach's α = 0.86). In this study, the scale demonstrated a high internal consistency (Cronbach's α = 0.92).

#### 4.3.3 Lifelong Learning Scale

The tendency of students to engage in lifelong learning was measured through the use of the Lifelong Learning Scale developed by (Kirby et al. 2010), adapted into Turkish by (Arslan and Akcaalan 2015). The scale is unidimensional and includes 14 items (for example, “I enjoy learning for its own sake”; and the reverse coded item: “I like to have others make plans for my learning”). All items were scored on a five-point Likert scale. The results of the validation study confirmed the scale was unidimensional and had acceptable fit measures (χ^2^/df = 3.89, RMSEA = 0.070, GFI = 0.94, SRMR = 0.059, and CFI = 0.89). Cronbach alpha coefficient of 0.84 indicated adequate internal consistency for the current study.

### 4.4 Procedure for collecting data

The research design for this study employed a cross-sectional, correlational and survey-based approach. Data were collected through an electronic survey hosted on Google Forms from late March to early May, 2024. Ethical approval for conducting the research at all four participating universities was granted prior to the start of the data collection process. The ethical standards outlined in the 1964 Helsinki Declaration and its subsequent revisions were followed during the conduct of this study.

The survey was distributed to a convenience sample of pre-service teachers in education departments of all four participating universities in Northern Cyprus (Kyrenia) through link distribution methods (QR codes, email lists, and student WhatsApp groups) by official university channels and courses taught by instructors at those institutions. An informed consent statement in digital form was displayed on the first page of the survey. Participants were informed regarding the academic purpose of the study, assured that their participation would be anonymous and confidential, and told that their participation was entirely voluntary. Informed consent was electronically obtained from all participants before they proceeded with completing the questionnaire.

The survey was divided into two distinct sections. Demographic information was collected in the first section and the three established scales measuring the two outcome variables were in the second section. The AI Anxiety Scale, the Multidimensional 21st-Century Skills Scale, and the Lifelong Learning Scale were in that order. The survey was available in Turkish and English. The survey was estimated to take 15–20 min to limit participant fatigue and prevent careless responding. Incomplete responses and straight-lining were checked in the dataset after the data collection process had been completed and prior to statistical analysis.

### 4.5 Data analysis

The data were analyzed using IBM SPSS Statistics Version 27. After excluding 14 invalid responses, a total of 396 responses remained for the analytical purposes of the study. The final dataset did not have any missing values. The analysis of data was conducted in two phases. In the first stage, simple linear regression was applied to examine the direct impacts of AI anxiety in predicting students' 21st-century skills (H1) and lifelong learning tendencies (H2). The second stage used multiple linear regression to examine how the four dimensions of AI anxiety differentially predicted 21st-century skills domains (H3). To determine the explanatory power of each model, *R*^2^ and Adjusted *R*^2^ values were calculated.

Prior to testing hypotheses, preliminary analyses were conducted, which included descriptive statistics, Pearson correlations, and reliability estimates (Cronbach's alpha) for all the variables of the study. The normality assumption was verified through examining skewness and kurtosis statistics for all variables, which were within the acceptable limits of ±2 (George and Mallery, 2010). The assumptions of linearity and homoscedasticity were also evaluated through visual inspection of scatter plots of standardized residuals, which indicated that both assumptions were satisfactorily met. Additionally, multivariate collinearity was assessed using the Variance Inflation Factors (VIF) for all predictors, none of which exceeded 5.0 (Hair et al., 2006), with corresponding tolerance values above 0.20.

To evaluate the potential for common method bias, Harman's Single-Factor Test was applied (Harman, 1967). The unrotated factor solution showed that the first factor accounted for 24.29% of the total variance, well-below the conventional threshold of 50%. Thus, the observed relationship between constructs did not appear to be influenced by common method bias.

All demographic variables were collected for the purposes of describing the sample, but were not entered as covariates into the regression models to ensure model parsimony and allow for an examination of the theoretical relationships among the psychological constructs.

## 5 Results

This section presents the findings of the regression analyses carried out to test the hypotheses in relation to the predictive relationships among AI anxiety, the 21st-century skills, and LLT among students.

### 5.1 Overall prediction of 21st-century skills (H1)

Hypothesis 1 (H1) was that the overall AI anxiety would significantly and negatively predict the overall 21st-century skills of undergraduate students. The analysis explored treating AI anxiety as a single composite construct. Simple linear regression was conducted with the composite AI anxiety score as a predictor and the composite 21st-century skills score as an outcome. The analysis indicated that the general predictive relationship was non-significant as shown in Table 1, *F*_(1, 394)_ = 0.107, *p* = 0.744. The model did not explain any significant variance in 21st-century skills (*R*^2^ = 0.000).

**Table 1:** Simple linear regression predicting overall 21st-century skills from overall AI anxiety.

Predictor	*B*	*SE*	β	*t*	*p*
(Constant)	3.897	0.082	–	47.463	< 0.001
AI anxiety	0.009	0.028	0.016	0.327	0.744

*N*= 396.

*R*^2^= 0.000, *F*_(1, 394)_ = 0.107, and *p*= 0.744.

The findings show that H1 was not supported (*p* = 0.744). When considered as one construct, overall Artificial Intelligence Anxiety did not have a significant predictive relationship with the composite 21st-century skills of students (β = 0.016, *p* > 0.05). This absence of a significant predictive relationship supports the disaggregation of AI anxiety into its particular dimensions, and justifies the differentiated dimensional approach examined in Hypothesis 3.

### 5.2 Overall prediction of lifelong learning tendency (H2)

Hypothesis 2 (H2) proposed that AI anxiety significantly predicts students' Lifelong Learning Tendencies. The analysis was conducted in two stages to evaluate the relationship at both the overall and dimensional levels.

#### 5.2.1 Overall AI anxiety on LLT (Simple Linear Regression)

A preliminary Simple Linear Regression was conducted to examine the predictive capacity of the overall AI Anxiety construct on LLT. The results are shown in Table 2.

**Table 2:** Simple linear regression predicting LLT from overall AI anxiety.

Predictor	*B*	*SE*	β	*t*	*p*
(Constant)	3.354	0.100	–	33.682	< 0.001
AI anxiety	0.038	0.034	0.056	1.120	0.263

*N*= 396.

*R*^2^= 0.000, *F*_(1, 394)_ = 1.255, and *p*= 0.263.

As shown in Table 2, the findings indicate that the overall predictive relationship was found to be not statistically significant (*p* = 0.263).

#### 5.2.2 AI anxiety dimensions on LLT (Multiple Linear Regression)

To test H2 conclusively, a Multiple Linear Regression was conducted using the four AI anxiety dimensions as simultaneous predictors of LLT. The full dimensional analysis findings are shown in Table 3.

**Table 3:** Multiple regression analysis predicting LLT from AI anxiety sub-dimensions.

Predictor	*B*	*SE*	β	*t*	*p*
(Constant)	3.341	0.102	–	32.907	< 0.001
Learning AI anxiety	0.060	0.039	0.090	1.549	0.122
Job replacement anxiety	−0.085	0.050	−0.161	−1.678	0.094
Sociotechnical blindness	0.016	0.056	0.029	0.284	0.777
AI configuration anxiety	0.064	0.038	0.131	1.686	0.093

*N*= 396.

*R*= 0.141, *R*^2^= 0.020, *F*_(4, 391)_ = 1.997, and *p*= 0.094.

As shown in Table 3, in line with the general model, none of the AI anxiety dimensions emerged as significant predictors of LLT (*p* = 0.094). According to both the overall and dimensional analyses, the results indicated that H2 was not supported. The generalized AI anxiety construct and its individual dimensions did not have a significant predictive relationship with LLT of students.

### 5.3 Differential prediction of 21st-century skills domains (H3)

Hypothesis 3 (H3) stated that the four dimensions of AI anxiety would differ in their prediction of 21st-century skill domains. Testing of the critical Hypothesis 3 utilized a series of five multiple linear regression analyses—one for each of the five 21st-century skills domains. Each of the four AI anxiety dimensions was entered into each of the five regression analyses as a predictor variable.

#### 5.3.1 Information and technology literacy skills

A multiple linear regression analysis was conducted to determine which of the four dimensions of AI anxiety predicted the ITLS. The findings are shown in Table 4.

**Table 4:** Multiple regression analysis predicting ITLS from AI anxiety sub-dimensions.

Predictor	*B*	*SE*	β	*t*	*p*
(Constant)	4.132	0.109	–	37.841	< 0.001
Learning AI anxiety	−0.100	0.042	−0.140	−2.400	**0.017** ^*^
Job replacement anxiety	−0.052	0.054	−0.092	−0.956	0.340
Sociotechnical blindness	0.071	0.060	0.120	1.176	0.240
AI configuration anxiety	0.045	0.041	0.086	1.111	0.267

*N*= 396.

*R*= 0.151, *R*^2^= 0.023, *F*_(4, 391)_ = 2.271, and *p*= 0.061.

Bold values and the symbol ^*^ both indicate statistical significance (*p* < 0.05).

The overall model was not statistically significant [*F*_(4, 391)_ = 2.271, *p* = 0.061], indicating that no significant predictive relationship was found between the four AI anxiety dimensions and the overall construct of ITLS. The model accounted for *R*^2^ = 0.023 of the variance. Although Learning AI anxiety emerged as a statistically significant negative individual coefficient (β = −0.140, *p* = 0.017), this predictor is not interpreted as the overall model did not reach statistical significance.

#### 5.3.2 Critical thinking and problem-solving skills

A multiple linear regression analysis was conducted to predict the Critical Thinking and Problem Solving Skills from the four AI anxiety dimensions. The findings are shown in Table 5.

**Table 5:** Multiple regression analysis predicting CTPS from AI anxiety sub-dimensions.

Predictor	*B*	*SE*	β	*t*	*p*
(Constant)	3.347	0.167	–	19.989	< 0.001
Learning AI anxiety	−0.305	0.064	−0.269	−4.767	**< 0.001** ^*^
Job replacement anxiety	0.160	0.083	0.178	1.918	0.056
Sociotechnical blindness	0.147	0.092	0.158	1.594	0.112
AI configuration anxiety	−0.048	0.062	−0.058	−0.768	0.443

*N*= 396.

*R*= 0.289, *R*^2^= 0.083, *F*_(4, 391)_ = 8.899, and *p* < 0.001.

Bold values and the symbol ^*^ both indicate statistical significance (*p* < 0.05).

The overall model was statistically significant [*F*_(4, 391)_ = 8.899, *p* < 0.001]. The model accounted for *R*^2^ = 0.083 of the variance in Critical Thinking and Problem Solving Skills. Learning AI anxiety emerged as a significant negative predictor (β = −0.269, *p* < 0.001). This finding indicates a significant negative relationship between Learning AI anxiety and higher-order cognitive skill development, providing support for H3 in this domain.

#### 5.3.3 Entrepreneurship and innovation skills

A multiple linear regression analysis was conducted to predict the EIS from the four AI anxiety dimensions. The findings are shown in Table 6.

**Table 6:** Multiple regression analysis predicting EIS from AI anxiety sub-dimensions.

Predictor	*B*	*SE*	β	*t*	*p*
(Constant)	3.814	0.130	–	29.416	< 0.001
Learning AI anxiety	−0.013	0.050	−0.016	−0.270	0.788
Job replacement anxiety	−0.171	0.064	−0.254	−2.645	**0.009** ^*^
Sociotechnical blindness	0.079	0.072	0.113	1.105	0.270
AI configuration anxiety	0.083	0.048	0.134	1.730	0.084

*N*= 396.

*R*= 0.153, *R*^2^= 0.023, *F*_(4, 391)_ = 2.336, and *p*= 0.055.

Bold values and the symbol ^*^ both indicate statistical significance (*p* < 0.05).

The overall model was not statistically significant [*F*_(4, 391)_ = 2.336, *p* = 0.055], indicating that no significant predictive relationship was found between the four AI anxiety dimensions and the overall construct of EIS. The model accounted for *R*^2^ = 0.023 of the variance. Although Job Replacement anxiety emerged as a statistically significant negative individual coefficient (β = −0.254, *p* = 0.009), this predictor is not interpreted as the overall model did not reach statistical significance.

#### 5.3.4 Social responsibility and leadership skills

A multiple linear regression analysis was conducted to predict the SRLS from the four AI anxiety dimensions. The findings are shown in Table 7.

**Table 7:** Multiple regression analysis predicting SRLS from AI anxiety sub-dimensions.

Predictor	*B*	*SE*	β	*t*	*p*
(Constant)	3.839	0.127	–	30.261	< 0.001
Learning AI anxiety	0.073	0.049	0.087	1.506	0.133
Job replacement anxiety	−0.239	0.063	−0.361	−3.795	**< 0.001** ^*^
Sociotechnical blindness	0.153	0.070	0.220	2.177	**0.030** ^*^
AI configuration anxiety	0.000	0.047	0.001	0.010	0.992

*N*= 396.

*R*= 0.197, *R*^2^= 0.039, *F*_(4, 391)_ = 3.954, and *p*= 0.004.

Bold values and the symbol ^*^ both indicate statistical significance (*p* < 0.05).

The overall model was statistically significant [*F*_(4, 391)_ = 3.954, *p* = 0.004]. The model accounted for *R*^2^ = 0.039 of the variance in SRLS. Job Replacement Anxiety emerged as a significant negative predictor (β = −0.361, *p* < 0.001). In addition, Sociotechnical Blindness emerged as a significant positive predictor (β = 0.220, *p* = 0.030). This finding provides support for H3, indicating that anxiety related to potential job replacement significantly and negatively affects students' social and leadership skills, while Sociotechnical Blindness emerged as a significant positive predictor of the same outcome.

#### 5.3.5 Career awareness

A multiple linear regression analysis was conducted to predict Career Awareness from the four AI anxiety dimensions. The findings are shown in Table 8.

**Table 8:** Multiple regression analysis predicting career awareness from AI anxiety sub-dimensions.

Predictor	*B*	*SE*	β	*t*	*p*
(Constant)	4.120	0.109	–	37.909	< 0.001
Learning AI anxiety	−0.121	0.042	−0.167	−2.901	**0.004** ^*^
Job replacement anxiety	0.023	0.054	0.040	0.425	0.671
Sociotechnical blindness	0.086	0.060	0.144	1.426	0.155
AI configuration anxiety	0.031	0.040	0.059	0.770	0.442

*N*= 396.

*R*= 0.208, *R*^2^= 0.043, *F*_(4, 391)_ = 4.419, and *p*= 0.002.

Bold values and the symbol ^*^ both indicate statistical significance (*p* < 0.05).

The overall model was statistically significant [*F*_(4, 391)_ = 4.419, *p* = 0.002]. The model accounted for *R*^2^ = 0.043 of the variance in Career Awareness. Learning AI anxiety emerged as a significant negative predictor (β = −0.167, *p* = 0.004). This finding provides support for H3, indicating that anxiety related to learning and mastering AI technology significantly and negatively affects students' confidence in proactively planning their future careers.

The results supported Hypothesis 3 for skill domains in which the overall model reached statistical significance, confirming the study's use of a differentiated approach in determining the prediction of 21st-century skill domains.

## 6 Discussion

The rapid integration of AI into education has moved beyond a technical adoption phase to presenting profound psychological challenges. In this context, the current study aimed to determine whether AI anxiety is a significant predictor of 21st-century skills and lifelong learning tendencies among pre-service teachers studying in the Kyrenia area of Northern Cyprus.

Regarding H1, it was hypothesized that composite AI anxiety would be a significant negative predictor of the 21st-century skills of students. However, this hypothesis was not supported by the results of the regression analysis. This finding warrants careful interpretation. It potentially reflects that treating AI anxiety as a single construct may oversimplify the “Digital Native” experience, wherein the overall generalized technology anxiety has been superseded by more domain-specific anxieties. Although prior research often assumes a linear and monolithic relationship between anxiety and skill inhibition (Salovaara and Tamminen, 2009), the findings contradict this conventional assumption. The non-significance of H1 indicates that for university students, the interface itself is no longer an obstacle; anxiety has fragmented into domain-specific concerns. This result is consistent with the conclusions of (Wang and Wang 2019) who suggest that an overall score of AI anxiety may obscure individual predictive pathways. By reducing AI anxiety to one construct, the differential impacts of its sub-dimensions, with some potentially substantial and others non-significant, effectively neutralize the predictive strength of the composite. This distinction is crucial: the general fear of technology may have been a detrimental factor for previous generations, but the skill development of the current generation is not limited by generalized technological anxiety, but rather by specific anxiety-inducing concerns, such as the complexity of learning or fear of future unemployment. The non-support of H1 therefore does not imply the absence of an effect, but rather points to the multidimensional nature of the relationship between AI anxiety and student outcomes in the contemporary educational environment.

For H2, the hypothesis was that AI anxiety would be a significant predictor of students' lifelong learning tendencies. However, this hypothesis was not supported because lifelong learning tendencies were not significantly predicted by AI anxiety. This result is particularly notable as it suggests that students' lifelong learning orientation is resilient to AI-related anxiety. This resilience is consistent with SDT, in which an intrinsic motivational orientation is protected against extrinsic technological pressures (Deci and Ryan, 2000). This finding implies that although AI anxiety can disrupt specific skill development, it does not diminish students' intrinsic motivation to learn. Consistent with (Åström et al. 2025) and (Shevchenko et al. 2025), these results suggest that students' intrinsic drive for learning is largely unaffected by AI-related threats. Even when students experience AI as a potential threat to their professions, their identities as learners is not easily destabilized by such external pressures. This is consistent with the view that an autonomous orientation to learning acts as a psychological buffer, enabling students to adapt to technological change without losing their commitment to growth (Yang, 2024).

H3, which predicted that specific sub-dimensions of AI anxiety would have differential effects on skill domains, was supported. The regression analysis showed a clear and theoretically coherent pattern of results consistent with the employed cognitive and motivational theories. Learning AI anxiety—reflecting the perceived difficulty of mastering AI—emerged as the primary barrier to cognitive skill development and a significant negative predictor of CTPS. This is consistent with CLT, which posits that the extraneous load from mastering the complexity of AI reduces the working-memory capacity available for higher-order thinking (Basanovic et al., 2018; Sweller, 1988). According to (Fan et al. 2025), students are not necessarily afraid of the technical interface, but their cognitive load is increased by the underlying logic of AI, and this consequently hinders their capacity to process and synthesize information critically. Learning AI anxiety further emerged as a significant negative predictor of Career Awareness, indicating that the demand of mastering AI technology constrains students' confidence in planning their professional futures. The analysis also revealed an independent motivational pathway where anxiety about extrinsic threats inhibited professional agency. Job Replacement anxiety emerged as a significant negative predictor of SRLS. Consistent with SDT and SLT, this finding suggests that when career prospects are perceived as threatened by automation, the motivation to lead and engage socially emerges as the most vulnerable skill (Bandura, 1977; Ryan and Deci, 2020). This professional threat is a potent extrinsic stressor that erodes perceptions of competence and autonomy essential for proactive leadership behavior. A paradoxical finding was the significant positive prediction of Social Responsibility and Leadership by Sociotechnical Blindness. This result suggests that students who score high on sociotechnical blindness simultaneously report social responsibility and leadership, pointing to a disconnection between leadership self-perception and awareness of AI's systemic implications. Such a result aligns with (Moores and Chang 2009) caution against algorithmic complacency, suggesting that leadership in the AI age should be grounded in ethical literacy to ensure that self-confidence does not come at the expense of ethical responsibility. This counterintuitive result may also be explained by the phenomenon of illusory confidence as described in the Dunning-Kruger effect (Kruger and Dunning, 1999). Students with limited awareness of how AI systems are socially and ethically embedded lack precisely the conceptual framework needed to accurately assess their own readiness for AI-mediated leadership. As (Dunning 2011) demonstrated, knowledge deficits in complex domains are structurally self-concealing—the same understanding required to perform competently is required to evaluate one's own performance accurately. In the absence of any meaningful referent for technological governance, students may register high leadership confidence not despite their sociotechnical blindness but because of it, as the complexity they cannot perceive makes no demands on their self-assessment. (Rozenblit and Keil 2002) work on the illusion of explanatory depth further supports this interpretation, demonstrating that individuals systematically overestimate their causal understanding of complex systems—a pattern directly applicable to student self-perceptions of AI-related leadership capacity. The finding may also reflect the fact that pre-service teachers in this sample conceptualize leadership primarily through the lens of interpersonal leadership rather than technological governance. Consistent with social identity theory (Tajfel and Turner, 1986), education students' leadership self-concept is shaped by the normative expectations of the teacher role—student advocacy, collaborative practice, and moral care for learners—all of which are relational and interpersonal in orientation. (Spillane 2005) has theorized teacher leadership precisely in these terms, and within this frame students' high social responsibility and leadership scores may reflect genuine and well-founded interpersonal competence. Technological governance—encompassing accountability for algorithmic systems, institutional AI policy, and the ethical oversight of automated decision-making—remains largely absent from this self-concept, not because students reject it but because teacher preparation programs have yet to establish it as a leadership expectation (Selwyn, 2019). Sociotechnical blindness does not produce leadership capacity; it leaves a critical dimension of leadership invisible within an otherwise coherent professional identity. The paradox can also be deepened by the presence of moral blind spots (Bazerman and Tenbrunsel, 2011)—conditions in which genuinely held ethical commitments coexist with systematic failures of moral attention in domains not yet perceived as morally salient. (Bandura 1999) theory of moral disengagement similarly demonstrates that individuals maintain strong prosocial self-images while remaining cognitively insulated from the ethical implications of domains they do not yet recognize as ethically demanding. Students high in sociotechnical blindness may be entirely sincere in their commitment to social responsibility while that commitment has not yet extended to the domain of algorithmic bias—the systematic embedding of discriminatory outputs in AI systems. This gap exists precisely because the ethical stakes of that domain have not been rendered visible within their education. The scholarship on algorithmic bias makes clear that such moral blind spots carry serious institutional consequences, as the harms embedded in algorithmic systems are most likely to persist where those responsible for oversight lack the sociotechnical awareness to identify them (Noble, 2018; O'Neil, 2017). Finally, the possibility of measurement artifacts must be acknowledged. (Paulhus and John 1998) identified moralistic bias—the tendency to self-enhance on communal traits such as social responsibility—as particularly pronounced among individuals whose professional identity is communally oriented, which is precisely the profile of pre-service teachers. The use of self-report instruments for both predictor and outcome variables further raises the possibility of common method variance (Podsakoff et al., 2003), whereby shared response tendencies such as social desirability may inflate observed correlations. Future research should examine whether this pattern persists when leadership is operationalized to explicitly include technological governance and algorithmic accountability, and whether interventions that render sociotechnical awareness professionally salient disrupt the illusory confidence the present data suggest.

Although several significant relationships between variables were identified, the explanatory power of the regression models was modest (ranging from 0.039 to 0.083). While these values are consistent with those reported in comparable studies of psychological predictors in educational contexts (Cohen, 1988), they indicate that the four dimensions of AI anxiety explain a limited proportion of the variance in 21st-century skills and LLT.

Several mediating and moderating variables were not included in the current models. AI self-efficacy, which refers to the belief in one's capacity to successfully perform AI-related tasks, is among the most likely candidates, given its established role as a mediator between anxiety and behavioral outcomes in technology-related contexts (Compeau and Higgins, 1995; Bandura, 1997). Digital literacy, defined as the ability to critically assess and effectively use digital tools and information, represents a further variable likely to moderate the relationship between AI anxiety and skill development, as students with stronger digital foundations may be better equipped to reframe AI-related challenges as manageable rather than threatening (Warschauer, 2004). Institutional support, including access to AI training resources, instructor guidance, and a psychologically safe learning environment, has also been identified as a contextual moderator of technology anxiety and academic outcomes (Hazzan-Bishara et al., 2025). These variables should be incorporated in future studies using mediated or moderated regression frameworks, or structural equation modeling designs, to provide a more comprehensive account of how AI anxiety shapes student competency development.

## 7 Conclusion

Students who perceive agency over AI logic and are confident about their future professional careers tend to demonstrate strong critical thinking, information literacy, and social leadership; in this state of psychological safety, the resilience threshold of lifelong learning tendencies remains intact, thus providing the ability to seamlessly integrate 21st-century skills into professional identity.

On the other hand, having to manage the educational environment with the burden of learning anxiety and the fear of being replaced in the job sector may harm students' self-efficacy in specific areas, including problem-solving, ethical leadership, and proactive innovation. This mental and professional distress, as the results indicate, is a specific inhibitor that drains the psychological resources needed to perform higher-order synthesis and diminishes student motivation in terms of professional agency. Consequently, the shift into an AI-enhanced period needs to involve not only technical training but also a psychological support system which will target these particular anxiety determinants to sustain academic engagement and institutional balance in higher education.

## 8 Implications

Regarding the theoretical implications of this research, findings suggest that emotional and socio-cognitive determinants emerge as the key motivators of students' attitudes and behaviors toward technology. Moreover, the relationship between sociotechnical blindness and inflated self-perceptions provides grounds for reconsidering how confidence in personal skills, potentially rooted in a limited critical awareness of technology, manifests in academic contexts. Overall, the findings indicate the need for theoretical models that explain the intricate relationship between anxiety, awareness, and skill development in educational settings. The implications for learning and teaching are practical and extensive. The technological competence and psychological resilience of future teachers can only be developed through a paradigmatic shift toward an integrated and holistic approach to student support. This will involve more than technical AI literacy training; it will require helping students develop psychological competencies, ethical reasoning, and engagement with realistic career pathways embedded within the curriculum, enabling them to respond constructively to the uncertainties posed by AI (Aytaç, 2022; Takil et al., 2022). Based on this, the results imply that interventions should be tailored to the specific dimension of anxiety being addressed. Learning AI anxiety may be reduced via scaffolded AI exposure embedded in coursework, enabling students to develop competence gradually rather than via abrupt exposure to unfamiliar tools without support or guidance. Job Replacement anxiety necessitates career-integrated curricula that explicitly reframe AI as a professional tool rather than a professional threat, combined with mentoring that reinforces students' sense of occupational agency. Sociotechnical Blindness anxiety requires dedicated instruction on the social, ethical, and systemic dimensions of AI, so that students develop informed critical awareness. AI Configuration anxiety may be addressed through intentional educational technology design and guided technical onboarding that reduces extraneous cognitive load prior to student engagement with AI tools for learning tasks. Addressing AI anxiety further necessitates that educators participate in faculty development programs that will enable them to address personal anxieties and provide an emotionally supportive learning environment that promotes digital competence and emotional resilience. Additionally, the sociotechnical aspects of AI must be explicitly integrated into the pedagogy; educating students on the ethical and societal implications of AI is essential to minimize sociotechnical blindness and cultivate critically informed dispositions necessary for future educators (Hopcan et al., 2024). Future studies need to employ extensive theoretical frameworks that clearly assess these mediating mechanisms. Also, qualitative studies can provide information about the psychological experiences of students, which support the different dimensions of AI anxiety and explain how each dimension affects the learning experience of students. Finally, given the cultural and disciplinary homogeneity of the present sample, the proposed model requires validation across interdisciplinary and cross-cultural contexts before broader generalizations can be drawn (Henrich et al., 2010; Hofstede, 2001).

## 9 Limitations

There are numerous limitations to this study that require additional investigation. One limitation of this study is the cross-sectional design that restricts causal inference and requires longitudinal studies to assess the temporal progression of the interplay between AI anxiety and skills. A further limitation concerns the composition of the sample. Although data were collected across four universities in Northern Cyprus, the sample predominantly comprised Turkish Cypriot and Turkish students enrolled in undergraduate education departments, with limited disciplinary and cultural diversity. Consequently, the findings cannot be assumed to generalize to pre-service teachers from different cultural backgrounds or to students from other disciplines (Henrich et al., 2010). Another limitation is that the given sample was restricted to pre-service teachers, self-reported 21st-century skill ratings are subject to social desirability bias. Future teachers may perceive these competencies as professional expectations and consequently overestimate their competence as opposed to what independent assessments might reveal.

## Data Availability

The raw data supporting the conclusions of this article will be made available by the authors, without undue reservation.
